# Social pathologies, recognition, and forms of life

**DOI:** 10.3389/fsoc.2025.1677696

**Published:** 2025-11-20

**Authors:** Aleksander Manterys

**Affiliations:** Faculty of Applied Social Sciences and Resocialization, University of Warsaw, Warsaw, Poland

**Keywords:** social pathology, recognition, forms of life, sociological theory, Axel Honneth, Vytautas Kavolis, Rahel Jaeggi, pathology of recognition

## Abstract

In this article, I consider whether and to what extent the concept of social pathology can serve to better understand relatively long-term disruptions of the social order. I compare the findings present in practically oriented studies on types of social pathology and attempts to explain it grounded in the body of sociological/social theory and social philosophy, serving the critique of society. I understand social pathology as a structured process. I try to indicate its conditionalities and to what extent the dynamics of this process affect the quality of social existence of individuals and collectivities. I show the emergence of relatively durable assemblages that “preserve” negatives in the form of pathological behaviors and their (often ideological) justifications. Seeking the possibility of theoretical synthesis in the thick approach formula, I indicate two positions that can be a starting point for further analyses: the concept of Vytautas Kavolis (set in the tradition of studies on social problems) and the concept of forms of life by Rahel Jaeggi (fitted in the formula of critical theory). I begin by recalling and criticizing the “classical” approaches to social pathology. Then, I reveal the connections between normativity and normality, and attempts to theoretically explain out-of-order phenomena. In the next part, I refer to the concepts of Kavolis and Jaeggi, portraying their relationships with critical theory. The final part is a tentative balance and an indication of issues worth taking up in the name of theoretical codification of knowledge about social pathology.

## Introductory remarks

This article can be conceived as another attempt to reactivate the concept of social pathology. In taking on this challenge, I attempt to accomplish several interwoven tasks. The first is to identify or reconstruct the contexts in which the term is used. I use the word “context” to emphasize that I intend to do more than simply enumerate the definitions of the term. This “something more” means presenting the “genealogy” of particular applications, which allows them to be embedded in a specific theoretical tradition, style of thought, or generalized discourse. It serves to understand what lies beneath the term, whether, how, and to what extent it is theoretically saturated, sometimes ideologically entangled, and what the intended and unintended consequences of its applications are. The second task is to consider conceptual alternatives or substitutes for the term “social pathology.” This primarily concerns bundles of associations and relationships with “social wrongs” or “social evils,” and more broadly, with what constitutes this or that “relatively enduring” variety of “social disorder.” The third task, a truly breakneck one, is to reflect on whether it is possible and advisable to combine threads drawn from different approaches.

I do not intend to add another gloss refining the findings in the field of pathologies of recognition, nor continue cataloging pathological-like “facts” reported by numerous textbooks on social problems. I refer to both of these narratives but somewhat instrumentally, extracting some of the threads and findings contained in them in order to specify the logic of the structured process of social pathology. The use of the term “structured process of social pathology” implies an attempt to outline the possibility of synthesizing findings “around” social pathology, transcending the horizons of particular theoretical approaches, or at least encouraging a revision of existing findings. Such a synthesis, or perhaps rather a minor “problem shift,” also serves—to reveal my own theoretical predilections—as a kind of commentary on the discussion surrounding the “interaction order,” emphasizing the importance of the dynamics inherent in social situations. These dynamics are structured, situated, as Goffman (1983) would say, not purely situational or contingent, because components of social structure and culture are present within them. For example, the situation in which domestic violence manifests itself involves not only the actors but also a confluence of conditions related to the situational configuration: from ritualized face-to-face relationships (among themselves, with neighbors, and with family), through socialization conditions, sometimes economic dependence, to the “pathological” reception of the institution of patriarchy and the suppression of criticism of its nature and consequences. Understanding this relationship requires a reconstruction of the interaction order. In particular, it needs identifying the circumstances or conditions behind the ritualized replication of actors' behaviors, the persistence of relationships, institutional arrangements, and cultural patterns, and—to express this again in the language of interactionism—the various accounts, rationalizations, excuses, or justifications.

I wonder whether and to what extent the concept of social pathology (along with the whole family of concepts addressing the negatives of social life) can serve to better understand disruptions, deviations, and threats to the social order. The discussion surrounding the various disturbances and perplexities of social life demonstrates the need for insights into various solutions, even if their theoretical validity may be questioned, as in the case of some early idiographic approaches to social pathology or quite contemporary textbooks enumerating areas of individual and/or social degeneration. To put it somewhat perversely, these solutions are addressed to various “publics.” The first are practitioners in the art of readaptation and rehabilitation of others. The second are those who understand social pathologies in terms of theoretical puzzles, the solution of which allows for illuminating the mystery of the relationship between the individual and society. The third are social philosophers whose reflections address human nature and the sources of social evil. I refer to the views of each of these “publics” not only to point out the strengths and weaknesses of the presented approaches but also to increase the chance of identifying those threads and findings worth pursuing in the name of codification and synthesis.

The task outlined in this way can be understood as an open declaration in favor of the broadest possible (thick) understanding of social pathology. The term “thick” refers to a certain unit-idea, present in Honneth's [(2000) 2007] work and taken up by Neuhouser (2023) and Harris (2019). The negative point of reference is “traditional liberal social criticism,” oriented toward “injustice” or “political-moral legitimacy.” This is because it closes the way to the analysis of numerous maladies that the narrowing lens of liberalism does not allow to see. This is a whole spectrum of phenomena that, regardless of whether they are “unjustice” or deprived of “political-moral legitimacy,” are threats to the existence of societies (e.g., global warming) or their individual and collective “segments” (e.g., colonial practices or cyberbullying). Coming down to the level of social situations, social pathologies constitute relatively permanent obstacles or barriers in achieving “the good life,” both on a daily basis and in subsequent interactional stages and various institutional arrangements. The point is to identify social forces inherent in the structural environment of actors, which prove destructive not as an interactional “cold,” but as chronic, transituational exposure to harmful (sometimes paralyzing) influences on human agency. Invoking the category of “good life” seems heuristically apt, as it directs the analysis toward determining what in the existence of individuals and collectivities is actually (and potentially can be) destructive in the sense of inhibiting or preventing the realization of their potential. As (Harris 2019, p. 48) notes, it is crucial to pay attention to “[…] a direct connection between certain forms of social existence and the capacity for human beings to attain the good life, however formulated.” Such a “normative orientation” of analysis constitutes an indispensable starting point for “ethical critique.”

The “thick” approach may be troubling, if only because the discussion around social pathology (and the entire family of related concepts) is, so to speak, uneven: dispersed, ideologically and theoretically diverse. This polyphony, if it is not to degenerate into cacophony, requires organization, a kind of “arrangement.” Remaining with this musical association, my own score contains several interwoven “motifs.” The first is the discourse around pathologies of recognition (or, more broadly, social pathologies in the sense of critical theory), whose findings, especially the move toward a thick approach, prompted me to take up this “topic.” The second is a foray into “conventional” sociology and the discussion around normality and normativity. The third is an indication of theoretical alternatives that, in my opinion, might be helpful in shaping the trajectory of a theoretical synthesis. To be clear, such a synthesis would be premature now, as prior selection and evaluation of the findings formulated within various approaches is necessary. The point is to select those that could be recognized as components of a universal approach to social pathologies, both in terms of the normative approach of “pathology diagnosis” and “ethical critique.”

I expand the thick approach “battlefield” by juxtaposing threads present in practically oriented (and usually theoretically ascetic) studies of types of social pathology, as well as theoretically grounded explanations of it, whether in the context of threats to the social order or as components of the critique of society. Such approach, in my opinion, encourages to look for common elements present in different narratives, regardless of their ideological and philosophical entanglements. By striving for the “theoretical saturation” of thick approach, I try to indicate objective conditionalities of social pathology, that is, those related to the functioning of societies, collectivities, and individuals. More precisely: I am interested in the relations between particular “molecules” of the life-process, whether and to what extent the structured dynamics of this process affects the quality of the social existence of individuals and collectivities. This allows, in particular, the characterization of emergence of relatively durable “assemblages” preserving negatives in the form of pathological behaviors and their justifications and rationalizations (also under the banner of ideological domination or hegemony). I use the term “assemblage,” I admit, somewhat intuitively and without any special affiliation with ANT categories, as a general term for those “relations” or “states of affairs” that tie heterogeneous elements together within a given event. The point is to focus on the dynamics of structuring processes, which is an inherent component of the definition of a situation, both in shaping a compatible flow and in creating a “seedbed” of turbulence. Considering the possibility of a theoretical synthesis of dispersed and often incommensurable findings, I indicate three theoretical solutions that fit into the thick formula approach. The first is Vytautas Kavolis's position (set in the tradition of studies on social problems), who “objectivizes” the concept of social pathology by referring it to the class of phenomena that are destructive to the self and others. The second is the critical theory of social pathologies, including pathologies of recognition. The third is Rahel Jaeggi's social theory, who conceives forms of life as basic and normatively marked instruments of adaptation to reality.

The roadmap is as follows. I begin by recalling the “classical” approaches to social pathology. I use quotation marks to stipulate in advance that they have little in common with what is considered “classical theories” in sociology. “Classics” in this sense refers to the time of their emergence and criticism, which in the 1940s and 1950s exposed the ideological nature of the seemingly objective (biologistic and medicalist) applications of this concept in the social sciences, and above all demonstrated their atheoretical nature and apparent practicality. Expanding the “battlefield,” I then show the connections between normativity and normality, which are the basis for formulating theoretical explanations of the occurrence of out-of-order phenomena. In the next part, I refer directly to the concepts of Kavolis and Jaeggi, showing at the same time their actual and potential connections with critical theory. The final part is an attempt to balance and indicate those threads or issues that are worth taking up in the name of theoretical codification of knowledge about social pathology.

## “Classical” terminology fields

The concept of social pathology in its “classical” application is a transfer of the biological or medical model to the analysis of, as it is often called, problematic phenomena of social life (Buchs, 2007; Lemert, 1951, 1972; Ryle, 1947). The point is not that we are dealing with a downright “naturalistic import,” which is done by analogy, homology or simply metaphorically, and sometimes “literally,” but whether in fact what is understandable (allegedly without special deliberations) on the solid ground of biology and medicine turns out to be heuristically fruitful in relation to phenomena that are the equivalent of disease, anomaly or abnormality in the domain of sociocultural phenomena. At first glance, the matter seems relatively simple: the approach of social pathology, as (Lemert 1972, p. 9–10) briefly recapitulates, is distinguished by the emphasis on the individual maladjustment, the impossibility or inability to successfully (or at least satisfactory) adapt to the requirements defined by the institutions of “conventional” society. In this sense, anything that deviates from the norms and morals that are in force at a given time can be considered as a social problem. Earlier approaches to social pathology (roughly the second half of the nineteenth century and the first decades of the twentieth century) are characterized by a reformist (and conservative) attitude. This attitude emphasizes the need for “treatment” (social rehabilitation/readaptation or resocialization), regardless of whether one is dealing with an overtly biological view of deviants as “born criminals,” or rather a narrative about an “infection” caused by the moral degradation of the deviant's environment. In a word, it postulates a decisive societal reaction to the abnormality of individuals and collectives (cf. Gillin, 1933; Lombroso, 2006; Queen and Mann, 1925; Smith, 1911; see also Dickson et al., 2020). Objects and procedures of this “resocializational” treatment determine the dominant worldview of well-informed citizens who, while supporting progress/development and driven by concern for the “health” of society, seem not to notice the pathogenicity of pro-development social dynamics and the structural inability to adjustment (or forced/constrained maladjustment) by large segments of the society's population.

There are several fundamental reasons why such a picture of social pathology, both in the common-sense and scientific sense, has proven to be questionable. First, as Mills (1943) points out, it was the product of the professional ideology of researchers (social pathologists), who were a relatively homogeneous group originating from small towns and farms in the USA. For this reason, they were interested in exploring what they themselves know and recognize as real and practical. Such an approach results in an atomized view of social life, while seemingly apolitical practicality turns into democratic opportunism. Second, the aversion to abstraction, generalized knowledge in the field of social pathology means the production of platitudes, reflected common-sense characteristics of social evil, and their reproduction in the form of numerous textbooks. This is tantamount to consistent theoretical abstinence, not undertaking the analysis of the relations between social pathology and structural conditions and norms (Mills, 1943; Sutherland, 1945). It means a permission for methodological anything goes under the banner of multi-factorial analysis. Third, the normative character of social pathology is neutralized by recognizing that any manifestations of social pathology are deviations from ethical-normative harmony. Any social change is *eo ipso* perceived as a threat to the social order or, paraphrasing Karl Marx, it shatters the idiocy of the idyll of rural life. The particular interpretation of this order, which itself is a product of the actors/agents of society, is not only recognized as universal, but is even sacralized. It is, in essence, a simple “mechanics” of static homeostasis, a rural-based caricature of biological organicism. It does not take into account the dynamics of interaction with the environment and the variability of the sociocultural environment, *ex definitione* conceives development and change as a mortal threat to welfare-state and particular wellbeings. Fourth, it ignores the processes through which particular individuals/groups, or their behaviors, come to be perceived in terms of deviance and pathology [Becker, 1963; Best, 2006; Goffman, (1963) 1986; Lemert, 1972; Kitsuse, 1962; Spector and Kitsuse, (2001) 2009].

This jeremiad, composed quite selectively, seems to have no end. One can discuss *ad libitum* the simplified view of subculture in terms of its opposition to mainstream or conventional culture or wonder why some youngsters from good families choose a life of crime, not to mention the “excellently-socialized” and well-adjusted business-sharks and banksters. From a theoretical point of view, it is important that the “classical” concept of social pathology loses its attractiveness in favor of other approaches to social problems, in terms of social disorganization (Blumer, 1937; Kramer, 1943; Markowitz et al., 2001; Shaw and McKay, 1969), dysfunction and anomie (Merton, 1968, chs. 6 and 7), stigmatization and labeling [Becker, 1963; Goffman, (1963) 1986; Link and Phelan, 2001], deviation (Adler and Adler, 2006; Clinard and Meier, 2011; Lemert, 1972; Goode, 2016) and reality construction [(Spector and Kitsuse, (2001) 2009)]. It should also be noted that the doubts and questions indicated above are still neutralized by the spell of practicality and the production of idiographic descriptions of social pathology. They take the form of narratives (sometimes fascinating) in the style of Oscar Lewis or streaming documentaries about hardened carriers of social pathology, or subsequent textbooks, supplemented with the characteristics of new threats to the social order, e.g., in the form of domestic violence, virtual reality, artificial intelligence, global warming. In short, they catch everything that disrupts social integration, at least in the eyes of researchers of the social cohesion “genre” (see e.g., Delhey et al., 2023; Fonseca et al., 2019).

Since the 1960s, a significant change has taken place in thinking about social pathology. First, the observation (this time in a liberal or radical version) that social institutions themselves are pathological, or their functioning is the subsoil of behaviors pathological. Either way, as (Rubington and Weinberg 2003, p. 15–19) state, the ultimate cause of social pathology is socialization deficits or failures, whether in the sense of defects inherent in the individual, or in the sense of acquiring “bad” values.

The roots of evil lie in genetical inheritance and social environment, or rather: regardless of which of them is considered more basic or primary, the researcher's task is to identify the causes or syndromes of “social diseases.” For example, (Horwitz 1984, 1990, 2007) shows the economic conditions of social pathology, a kind of syndromes of long-term economic deprivation, connections with changes in social control, and finally the transformation of normality into pathology decreed by the *DSM*, when it comes to recognizing normal emotions as manifestations of disorders or dysfunctions.

“Classical” approaches to social pathology are relatively easy targets for criticism. Their founding sin is reducing the complexity and dynamics of social life to the idiography of cases arbitrarily deemed evil or harmful. The struggle for social health is a story about individuals and collectivities doomed to failure, a chronic lack of success in taking conventionally expected social roles. This vision of the world and human nature is an Enlightenment caricature of the fatalism known from Greek tragedy. In the ancient original, the reader/spectator perceives the forces behind the actions of specific *dramatis personae*, understands what is *comme il faut* and why, and what is evil and why. He/she sees the consequences of adopting a specific definition of a situation, choosing a specific course of action within the context of the relationship between the present, the future, and the past. The narrative of “professional social pathologists” boils down to defining an individual or collective anti-hero/in, the carrier of this other social disease, and what to do with him/her. This story, for at least several reasons, turns out to be unconvincing.

First, although the object of study is the “factual” maladies that plague individuals and collectivities, their research does not lead to generalizations that go beyond the confines of individual cases. There is a fundamental difference between even an ultra-detailed description of a phenomenon and an attempt to understand or explain it. The point is not to anchor it in specific cases, but to formulate an answer to the question “why.” A suicidologist does not have to stop at ascertaining the multitude of causes and conditions behind every successful or unsuccessful attempt to take one's life. He or she can, as Durkheim [(1897) 1993, books 2 and 3] did, identify types and subtypes of suicide and point to their distinct causes. The explanation of this social fact is embedded in the logic of demonstrating the connections between the individual and society and referring to what is happening within society itself. The individualizing focus of “classical” social pathologists and their emphasis on the description of individual cases essentially establishes a division between the normal and the abnormal (or pathological) in relation to the reality being studied. (Thomas and Znaniecki 1918, p. 9), tracing the processes of social disorganization through the adaptive perturbations of Polish immigrants in the USA, clearly declare that “There is no break in continuity between the normal and the abnormal in concrete life that would permit any exact separation of the corresponding bodies of material, and the nature of the normal and the abnormal as determined by theoretical abstraction can be perfectly understood only with the help of comparison.”

Second, referring to (Becker 1963, chs. 1 and 2), “classical” concepts of social pathology conceptualize acts of deviance as symptoms of social disease. The lens of “disease etiology,” transferred to the discourse of social science, views normality as taken for granted. It simultaneously conceives it as a homeostatic touchstone for evaluating human actions. Consequently, it blurs the differences in beliefs about “healthy” behaviors that occur within society. Moreover, the systematics of “social diseases” means isolating their characteristic “symptoms,” or factors of social pathology. The use of multivariate analysis assumes “[…] that all the factors that operate to produce the phenomenon under study operate simultaneously” (Becker, 1963, p. 22). Such a “simultaneous” approach, in a sense, “stabilizes” the subject of study, but at the same time reduces the dynamics of social life to a series of static insights. Not all factors associated with a given pathology are present at a given time. As (Becker 1963, p. 23) states, “[…] we need a model that takes into account the fact that patterns of behavior *develop* in orderly sequence.” Becker attempts to identify these behavioral patterns in relation to each stage of an individual's “career.” This is a structured process of becoming a deviant. For example, a person with access to marijuana decides to “try it” in a situation of alienation from the norms of conventional society and with a focus on experimenting with his/her own experiences. Becoming a “declared” marijuana user (and at the same time an object-carrier of “undisputed” symptoms of social pathology) involves passing through successive stages (see Becker, 1963, especially chs. 3 and 4). Each of these stages constitutes a different interplay of social structures and personal motivations and aspirations. And it does not happen inevitably: a marijuana-user's career can be halted, not develop into a behavioral habit, at virtually any stage, but for various reasons.

Thirdly, the above-mentioned manifestations or aspects of theoretical abstinence, both in relation to the statics and dynamics of social life, are not mere “accidents at work,” but the fundamental motive driving the reproduction of atheoretical insights or examinations of certain aspects of social disease. (Sutherland 1945, p. 430) firmly rejects such an approach, clearly articulating the need to develop “[...] generalized knowledge in the field of social pathology, integrated with general knowledge in sociology [...].” A consensus around the term itself, its obviousness (assumed at the turn of the nineteenth and twentieth centuries), based on associations “with maximum happiness or the general social welfare,” is not enough. This is not a purely “verbal” issue “[...] since the definition of any class of phenomena in a theoretical system must ultimately be coextensive with the theory which explains it” (Sutherland, 1945, p. 430–431). Best (2006), referring to both Mills (1943) and Sutherland (1945), states that the term “social pathology” in its “classical” meaning has little analytical value because “[...] its proponents could not agree on a workable way to define the concept. On the one hand, the term implied that it referred to an objectively definable set of social conditions—those phenomena that could be recognized as diseases of society. Yet, on the other hand, the identification of social pathologies proved to be highly subjective, dependent upon the interests of those members of society who identified some conditions as pathological and upon the prejudices and presumptions of the authors who selected topics for inclusion in their texts.” Consequently, the heuristic potential inherent in the concept of social pathology is nipped in the bud and does not lead to the development of a (better) theory that could be competitive with alternative approaches. It does not lead to the identification of common elements within the phenomenon of social pathology and/or its various types. It stifles imagination and sensitivity to the “non-obvious” maladies of social life, nor does it lead to the emergence of new “puzzles” or reflection on the real sources of particular social diseases, not to mention a critique of the *status quo*.

Fourth, the conviction of the cognitive and research impotence of the “classical” perspective on social pathology leads some researchers to abandon or significantly modify it. There is undoubtedly a component in this movement away from the term “social pathology” conditioned by the whims of intellectual fashion, perhaps even political correctness. However, the fact that concepts of social disorganization or theories of deviance have pushed “classical” approaches to social pathology into the corner is primarily a result of their lack of any theoretical unity. For example, theories of social disorganization are currently a broad stream of research on the relationships “between place and crime.” They encompass a broad and constantly evolving spectrum of phenomena and analysis of types: from social ties and their effects, through determining the role of cultural factors, the impact of formal and informal social control, to ethnographic knowledge of how residents perceive their own neighborhood conditions (see Kubrin and Wietzer, 2003).

(Lemert 1951, 1972) gradually abandoned addressing sociopathic behavior in terms of social pathology in favor of tracing the influence of social agents (audience) on identity formation. This is a genuine theoretical puzzle that stimulates a whole series of insights into the modus “social control leads to deviation.” Characterizing this structured process reveals how law (reinforced by agents of control in society) is responsible for the escalation of minor rule violations. As (Lemert 1972, p. 63) explains, “Secondary deviation refers to a special class of socially defined responses which people make to problems created by the societal reaction to their deviance. These problems are essentially moral problems which revolve around stigmatization, punishments, segregation, and social control. […] They become central facts of existence for those experiencing them, altering the psychic structure, producing specialized organization of social roles, and self-regarding attitudes.” This scenario aims to establish the conditions or circumstances under which normalization of behavior (i.e., role adaptation) occurs. Key elements of normalization are labeling and self-labeling, and sometimes stigmatization, which persist even in “cured” and “normalized” drug addicts and alcoholics. In short, this narrative, in terms of “failured socialization,” reveals the “forces” behind the transition from primary to secondary deviance. In this narrative, a special role is played by the audience, whose agents, as Becker (1963) explicitly puts it, apply established rules (norms) to individuals and collectivities. These agents of control are “moral entrepreneurs,” holders of key power resources, who create deviance. They select those whose behavior they deem “offensive” and label them. That is, “[…] *social groups create deviance by making the rules whose infraction constitutes deviance*, and by applying those rules to particular people and labeling them as outsiders. From this point of view, deviance is not a quality of the act the person commits, but rather a consequence of the application by others of rules and sanctions to an ‘offender”' (Becker, 1963, p. 9). The ability to contest imposed definitions encompasses a wide range of procedures and reveals the determinants of the interpretive process, which is shaped by position in the power hierarchy. For example, a petty shoplifting incident may go unreported due to the strong position of the parents or be treated as a “juvenile incident.” However, if it is not neutralized in any way, it triggers the entire machinery of social reaction and has consequences for the “perpetrator's” self-image. It modifies his/her master status in terms of deviance, diminishing other components of the self, and often activates the mechanism of self-fulfilling prophecy. Consequently, it limits opportunities, encourages the selection of illegitimate forms of activity, and pushes toward a retrospective interpretation of his/herself and his/her deeds. It can lead to the “discovery” of new deviance, the strengthening of deviance, and the acceptance of the deviant label. To recapitulate: each of the above-mentioned approaches indicates the origins of outsiders, and thus presents structured processes for generating and maintaining areas considered social diseases or maladies. This knowledge is essential to ensure that the practical aspect of the science of society does not reduce itself to magic or simple “ordering-and-forbidding,” as (Thomas and Znaniecki 1918, p. 3 ff.) warned against.

The genealogy of social pathology turns out to be relatively winding or complicated. To put it simply: while on the basis of narrowly profiled studies of social problems (also in criminology) the concept of social pathology loses its analytical and research appeal, through the effort to embed it in the body of sociological/social theory and social philosophy it does not lose its significance, and even, so to speak, “gains new life.”

## Normality and normativity

Theoretical insight into the phenomenon of social pathology reveals a universalist intention to identify and analyze states of affairs considered as out of order, which are—as Douglas [(1966) 1984] used to call it—polluting and dangerous, posing threats to the social order and the existence of individuals and collectivities. The analogies related to the functioning of the mechanism and the organism, characteristic of Émile Durkheim's narrative [Durkheim, (1893–1902) 2013, see also Besnard, 2005], lead, perhaps contrary to Durkheim's own intentions, to the privilege of normality and social cohesion. In Talcott Parsons's systemic interpretation (Parsons, 1951, especially ch. 7, Parsons, 1964) lack of order is a threat to the system and its subsystems. Both of these (even paradigmatic) approaches see change or differentiation as something natural in the sociocultural context of action. Adjustment and adaptation are conceived as the achievement of states of dynamic equilibrium, and not as a mechanical “fit” or simple expression of the body's functions. The Parsonian conformist is not an opportunist or a cynic, but an actor making a decision about this or that action taking into account the available conditions and norms in a given situation. It can be said that pathological behavior is the result of a lack (failure) of proper coordination between socialization (social and cultural learning) and personal experiences, which is associated with inappropriate values, choices and mental states. Of course, the impact of social constraints is overwhelming, but it is the acting subject as a moral human being who “tunes” the engine that drives his/her choices and actions. This special relationship between normality and normativity is explained by Canguilhem [(1966) 1991; see also Gane, 1998, 2002; Margree, 2002]: in the order of facts, no object, phenomenon or process is either normal or pathological in itself. Anomalies, mutations, or—more broadly—all processes of change can be considered as manifestations of other forms of life. Thus, the source of normality is normativity, if only because other forms of life, in a different environment, can be considered as normal. The relationship between the functional norms of a living organism and the norms related to the conditions in which these norms are considered normal is important.

On the one hand, thinking in terms of functions and dysfunctions, especially in Robert K. Merton's interpretation (Merton, 1968, chs. 3, 6, and 7), taking into account manifest and latent functions and an attempt to answer the questions: “functional for whom” and how anomie manifests itself, is still useful in relation to social pathology (deviation). It conceives social pathology as a complex and dynamic phenomenon and enables its “addressing” both in relation to individuals and collectivities, as well as social institutions. Adaptative dysfunctions are somehow individually anchored, although at the same time they constitute attributes of action in a group, collectivity, in a specific institutional setting. They are associated with the “unsuccessful” (failed) internalization of values, including what is considered as right or adequate in culturally and structurally “marked” situations of action. On the other hand, even if one passes over in silence the “eternal” accusations of preserving the *status quo* and an oversocialized vision of human nature, it may not be sufficiently emphasized that some of the “incriminated” dysfunctions may have their source in the persistent pursuit of equilibrium and “normalization.” Paradoxically, society, through its institutions, “produces” deviants, if only because without them any change and development would be impossible. At the same time it incurs a cost by reproducing existing social pathologies or creating new ones when it tries to combat them or “solve social problems,” including those that it itself had previously generated. As Erikson (1966) put it directly, concern for the cultural integrity of the community depends on acts of deviation, it serves to maintain cultural identity, the integration of society, which is achieved by, *inter alia*, thanks to the criminal justice [see also Foucault, (1999) 2003]. However, as Reiman and Leighton [(1979) 2023, ch. 1] comment, this system actually creates a “reality” of crimes and criminals. It not only represses, but also has an ideological function, serving to maintain the position of the powerful at the expense of weaker individuals and collectivities. It is the Pyrrhic defeat, because social solidarity depends not on success in combating crime, but on failure to limit it.

The Erich Fromm's concept of “pathology of normalcy” radicalizes this way of thinking by pointing out the mechanism of consensual validation, strengthening or consolidating “socially patterned defects”—pathological conditions or circumstances that constitute a deviation from the “normative humanism” [Fromm, (1955) 2002, (1991) 2010]. More precisely: at the root of this sinister in its social and individual consequences process are deficits inherent in society, which prevent social subjects from realizing what is associated with a healthy, meaningful and happy life in terms of normative humanism. What is pathological, both in the dimension of everyday practices and the pathology of institutions of social life, is not perceived as a manifestation (and usually a product) of insane social life, the dehumanizing effects of objective social reality, but—as a socially patterned defect—becomes a norm of conduct and a touchstone of what is considered proper. In a word, it becomes a component of social and pathological normality at the same time. And it is worth adding that such an approach, although it “works” particularly well in relation to the alienating capitalist socio-economic formation, can just as well be—since Fromm's narrative is also a story of “mutual slaughter”—written as a generalized discourse not only about suffering, but also about how evil becomes a virtue on a mass scale, blocking the potential of individuals and collectivities. What is more, it not only deprives the quality of their social existence, but by alienating, it encourages the recognition that all societal and social maladies are normal, even natural. In a quite contemporary edition, this passes into the ruling of the “pathology of normality,” the sinister connection between the anti-humanist properties of capitalist social relations and cultural institutions. The main research task is to identify the relations between social pathology/ies and the cycles of domination and violence produced and reproduced in society. This leads directly to the question of the possibility of emancipatory transformation, mitigation or even abolition of the circumstances that cause oppression, exploitation and alienation (see especially Smith, 2017).

## Social pathologies, recognition, and forms of life

In the following sections, I will explicitly address three narratives that (in my opinion) are heuristically promising: Vytautas Kavolis's position, critical theory, and Rahel Jaeggi's social theory. Each, in its own way, goes beyond purely terminological findings and the simple symptomatology of out-of-order phenomena. The definition of the term “social pathology” occurs through, roughly speaking, an attempt to establish the circumstances in which a certain misdevelopment (out-of-order phenomen) is defined in terms of social evil or social wrong.

Kavolis, a researcher rooted in the tradition of studying social problems, attempts to reactivate the category of “social pathology,” clarify its meaning, and embed it within the body of sociological theory. This is also a “positive” response to the weaknesses of the “classical” approaches I noted earlier. By further defining the term, Kavolis maps the phenomenon of social pathology within the broader context of its connections with social organization and culture. He highlights the importance of moral cultures and their connections with the “logics” of evil. The characterization of the constitutive domains of social evil or social wrong, as presented by Kavolis, prompts analysis in terms of social practices that generate the emergence and persistence of social pathologies. I find a similar line of thought in critical theory, also in its orientation toward the moral and ethical determinants of wellbeing. Furthermore, addressing the disturbances of the life-process, “immanently” present in Kavolis, allows for tracing the structured processes of coping with social evils/social wrongs. Jaeggi's concept, in turn, allows for an understanding of the functional-normative nature of forms of life and the complexity of constructing lines of human action within the problem-solving framework. The question of when and why a specific form of life fails (does not operate), also in the collective dimension, leads to an analysis of the functional and ethical “ineffectiveness” of social practices.

### Destructive power of social pathology: Vytautas Kavolis' approach

Kavolis (1969) specifies and “objectivizes” the concept of social pathology by stating that it defines a class of phenomena that are destructive to the self and others. More precisely: the field of social pathology includes both behaviors that are destructive to the selves of individuals and their social and cultural conditionalities. This specific combination of, so to speak, pathomorphology and pathogenesis allows for indicating phenomena that can objectively cause “costs” in the form of “destruction of life, health, or sense of personal identity” (Kavolis, 1969, p. 3). Moreover, social problems—distinguished as social arrangements evaluated by their destructive human costs—cannot always be captured in terms of deviance, because not all deviant behaviors have destructive consequences or can be considered as socially undesirable. They also elude public awareness, when a social problem is defined as a state of affairs that social activists consider as requiring amelioration (Kavolis, 1969, p. 2).

According to (Kavolis 1969, 2 et seq.), the inclusion of social pathology in the domain of objective phenomena of destruction and self-destruction allows to judge its occurrence regardless of whether and in what ways it is institutionalized, culture-bound, related to tradition or morality or made conscious by individuals and collectivities. It also does not assume a violation of social stability or a sharp conflict. Pathogenicity and pathological behaviors are universal phenomena occurring in all human societies. Social structures and processes are not “normal” in themselves, or “pathological.” Specific circumstances, social arrangements become pathogenic to the extent that they lead to destruction or self-destruction in the domain of life, health, or the sense of personal identity. The fundamental task of the researcher is not so much to offer atomized or partial views of pathogenicity and pathological behaviors, but to indicate particular, so to speak, sociocultural assemblages that may constitute “fodder” in the process of emergence of behaviors resulting in destruction. The point is to determine whether and to what extent each of these pathogenic “assemblages” actually evokes or even induces individuals to undertake (“choose”) pathological behaviors in everyday situations of action and/or in specific institutional environments.

The advantages of this approach to social pathology are as follows. First, it covers a relatively broad, though clearly defined, range of destructiveness: from self-destructiveness, when an individual destroys his or her own life, health, or sense of personal identity, and other-destructiveness, when his or her actions have a destructive effect on the life, health, and identity of another person or persons. Second, the behavior pathological may be the “choice” of the individual, i.e., manifestation voluntarism (spontaneity) of his/her action, or to be an ingredient organized pathology “[…] when the individual is either ‘morally' obligated or ‘politically' coerced by the group or organization to whose authority or power he is subject to commit destructive or self-destructive acts (or to encourage others to commit such acts)” (Kavolis, 1969, p. 4). Thirdly, both of the above typologies serve not only to categorize entities within pathogenic conditions, but also (and above all) allow for the separation of possible types of relations between them. Fourthly, each of these categories, whether “subjective” or “relational,” included in the spectrum of analysis of pathogenesis and pathological behaviors, finds its application in various environments, different types of cultures and societies. It finds, to add following (Kavolis 1969, p. 5), as long as “In studying social pathology, we aim at establishing the characteristics of social structure and process as well as those of cultural orientation which promote pathological behavior wherever they occur.”

The study of social pathology becomes part of a broader project encompassing the analysis of the connections between pathological behavior and social organization (systems: family and kinship, stratification, economy and community), social change, culture, social control, as well as the phenomenon of secondary pathology, which develops as a reaction to the primary (i.e., “existing”) pathology. Encouragement for cross-cultural and trans-historical studies is obvious, because in this way it avoids the limitations of atheoretical, ideologically burdened and atomized approaches to the sources, course and consequences of individual destructiveness. The program of research on social pathology articulated by Kavolis does not eliminate deviance perspective, but even complements or strengthens it by indicating the origins of social pathologies (Kavolis, 1969, p. 5–38).

The development of this program is the specification, as (Kavolis 1977, 1985a,b) calls it, of the “family” sphere-of-life cultures, particularly scrupulous in relation to the moral cultures and logics of evil defined by secular moralities. It is about analyzing the main sociocultural alternatives that occur in specific spheres of human life, among which moral cultures function. It is necessary to identify and analyze those particular constellations of emotions and ideas that “mark” human action as right *resp*. wrong, in terms of responsibility and constraints. One can speak of a certain machinery of reinforcement good behaviors. They are conceived as moralization systems that determine the degree of conformism within specific moral constellations. Their characterization requires understanding their social context and components of their dynamics: “moralities of the moment,” “moods,” “energies”—relating to particular moral cultures which “frames” particular actions (Kavolis, 1977, p. 331–332).

The next conceptual re-entry, as to use Luhmann (1993) term, is the sacred–secular distinction. The issue is whether what is forbidden, what leads to suffering, i.e., refers to harm done to other persons (or collectivities), is defined in terms of obligations and sanctions toward what is supernatural and immutable (the sacred), or whether it refers to moral culture, which is the product of human actions (the secular). This distinction does not exclude “mixed” forms of moral cultures, and since it is to be applied universally, it does not “assign” the feature of secularity only to modernity (Kavolis, 1977, p. 332). Secular moral cultures of modernity may come close to “sacred” type cultures (e.g., ascetic-revolutionary moral culture) or, like liberalism, to preserve the sacralized rudiment in the form of inalienable rights. They can also directly transfer the mechanism of harmonization and conformity to institutions, reducing human agency. Moreover, in the historical process, there are “moral struggles” both between cultures and within a single moral culture, as well as in the intercultural dimension (Kavolis, 1977, p. 338). Each of the historical variants of moral cultures can be subsumed under more general logics of establishing, accepting and enforcing conformism. The first of them is the logic of establishing obligations, which sets out a genealogy of moral obligations: starting with personal moral intuition and ending with the universalizing group *noblesse oblige* (custom) or utilitarian reciprocity (Kavolis, 1977, p. 338–339). The second is the logic of justification of claims, which identifies certain goods and services as well as elements of symbolic recognition as components of what “[…] can be justified (made ‘rightful')” (Kavolis, 1977, p. 339). The third is the logic of moral decision-making, i.e., indicating the agency authorized to make decisions, behind which there is, as it were, a “moral self-reliance” (e.g., in the form of an oracle, a person with moral intelligence or mechanized pattern discernment). The fourth is the logic of moral evaluation, defining criteria/methods of evaluating actual behaviors (Kavolis, 1977, p. 340–341).

Such discernment in collective moral structures is essential, as it allows to lead the analysis toward recognizing and identifying the sphere of evil, usually in opposition to what figures as the good in the sociocultural space. This is the more it is important that contemporary secular moralities (like sacred moralities, earlier and modern) include “[...] total schemes for interpreting the meaning of existence within which evil has and assigned place” (Kavolis, 1985b, p. 189). The term “mythological paradigms” in relation to the understanding of evil combines seemingly incommensurable components of different narratives (from popular religion to literary imagination). It is the primary framework for interpreting evil and the basis for orientation in the world of everyday life. Subsequent re-entry leads to the emergence of “a variety of *logics* of evil, sets of assumptions about the causes of particular evils in human action or in nature, and about the best manner of responding to them in particular situations” (Kavolis, 1985b, p. 190). Logics of evil, unlike moral logics, are modalities of thinking-in-action, i.e., practices serving management, strategizing—to refer to Goffman (1969) or Schelling ((1960) 1980)—one's own actions. The point is not to maintain some pattern taken from the pool of this or that civilization, but to manage one's own affairs as best as possible. It is rather a balancing act, even a maneuvering in a series of practical achievements (Garfinkel, 1967) or in the spirit of Goffman being-on-the-wire [Goffman, (1967) 1982].

As (Kavolis 1985b, p. 190) contends, “In describing each logic, one must therefore take into account its basic cultural form, the phases of its historical development, and its alternative versions, as well as mixes, overlaps, and confusions between different types of logics.”

The “old” or “traditional” logics are not so much eliminated as placed on a kind of hierarchy of importance or—to use Schutz's (1970) term—in the system(s) of relevance. The decidedly secular liberal logic sees the sources of evil in the deficiencies of reason. In accordance with the “composite” nature of each logic of evil, it concerns both insanity as a state describing someone's personality, deficits related to ignorance, prejudice and moral cowardice, as well as restrictions on freedom. Accordingly, the remedies are psychiatry, universal education and the democratic rule of law (Kavolis, 1985b, p. 191). The technocratic logic considers that “[…] bad effects arise from inefficiencies or technical failures (‘accidents') of human or mechanical organization or from ‘traditionalist' or ‘gratuitous' resistances to the establishment of efficient organization” (Kavolis, 1985b, p. 201). Therapeutic logic understands evil as a disturbance of normality that results in “a deformation of normal spontaneity” or freedom (Kavolis, 1985b, p. 194–195). Naturalistic logic, on the other hand, sees the sources of evil in the “nature” of individuals or groups, their inclinations, which can be institutionalized in various types of social organization. Evil may have a strictly biological basis, e.g., intra-species aggression, to use the favorite example of evolutionary psychologists; it may also be a manifestation of the “exploitation” drive in relation to others and the environment; or, finally, be a contingent combination of many different natural factors (Kavolis, 1985b, p. 198).

The interpenetration of these logics (Kavolis, 1985b, p. 204–208) directs the analysis primarily to whether and to what extent each of them becomes relatively autonomous and somehow assigned to certain spheres of life (e.g., from the liberal to the legal), which resembles Luhmann (1982) approach to the reduction of complexity. More precisely, what makes the selection of this or that subsystem of the logic of evil prevail in certain social situations and in others become a function of common contingency? What are the degrees of freedom of the actors in the selection or choice of these or other logics of evil and—to call Goffman [(1974) 1986] term—within their frames? Is the mechanism of rationalization, of ceding to a few, sometimes anonymous technocracy, power over the decision-making process about what is good, right, normal, and so on, and in what circumstances? What are the most “adequate” types of publics, to refer to Burawoy (2005), for particular logics of evil? Which individual, collective, and institutional agents have the opportunity to effectively implement the components of particularistic logic in their own actions? Which agents become “fixed” on the opposite side of the mirror of goodness and normality, somehow rightfully forced into the ruts of evil and pathology? What are the possibilities of revitalizing traditional morality and re-sacralizing segments of everyday morality? Is the concept of evil morally rooted, and in what circumstances does it become a function of the “amoral” perception of someone's deeds? And finally: is it the case that nowadays, as (Kavolis 1985b, p. 205) alludes to, referring to the spread of mass culture, we are dealing with the emergence of a new logic of evil, embedded in a kind of connection (or evolutionary development) of mass culture with virtual reality?

One thing can be stated with certainty, regardless of the assessment of Kavolis's individual findings: his research program does not “fit” the generalized “classical” discourse on social pathology. (Best 2006, p. 534–537) skillfully reconstructs this “classical” discourse, labeling it as chronically lacking theoretical unity, analytical and research utility, and trivialized in the form of numerous textbooks. Kavolis approach does not fall under such criticism because—first—his approach to social pathology, combined with the analysis of moral systems/cultures and the logics of evil, is embedded in a broader program of studies on society, culture, and civilization (see especially Kavolis, 1985a). Second, the central categories for analyzing the “negatives” of human existence are quite precisely defined and assigned to specific levels of cultural and social organization. Thirdly, and finally, the emphasis on the “abstractization” of basic categories of analysis is accompanied by a conviction, even an order, of comparative analyses. It is about establishing what is universal in nature, as well as what is civilizationally/culturally specific.

Does it mean—to use the term (Laitinen and Särkelä 2019, p. 83)—that the treatment of social pathology “as an umbrella term for social wrongs” can lead to a fatalistic understanding of atheoreticality due to the researchers' orientation toward “critizable social arrangements?” Not necessarily, I think. (Piotrowski 2006a, p. x), whose position Laitinen and Särkelä consider it as downright emblematic in terms of the “incriminated” umbrella term, defines social pathology “[…] as social problems that are present in common consciousness, and are perceived as detrimental and destructive to individuals, groups, or the entire society.” Developing a single working definition of social pathology, both objective and universal is in principle impossible. However, this does not mean theoretical abstinence, but definitional restraint and redirecting the mainstream of research activity toward “[…] *understanding* of social pathology phenomena—their causes, mechanism, and social costs” (Piotrowski, 2006a, p. x). (Piotrowski 2006a, p. ix–x) justifies this way of proceeding by observing an unprecedented acceleration of changes taking place in almost every area of society. And consequently, he notes a proliferation of destructive acts, the more severe the greater the global effects of scientific and technological achievements, or more directly: the more powerful the possibilities of harming others and oneself become on every level of social life. This is one thing. The other is an open declaration in terms of social constructionism when Piotrowski assumes that social reality is constantly and anew constructed, along with what is considered pathological. In this sense, the reconstruction of the genealogy of constructing social problems or social pathologies (or social evils/wrongs) by meticulously presenting the processes “of confrontation of perspectives and definitions of individuals, institutions and social groups,” seems quite reasonable and theoretically embedded. Proponents of grounded theory would probably recall the category of theoretical saturation and the procedure for moving from substantive to formal theory. Old-school mainstream sociologists would point to the possibility of creating middle-range theories in relation to specific types, genres, and subgenres of social pathology. It can also be argued that thematically and methodologically diverse texts included in the book edited by Piotrowski (2006b) attest to the need for inter- or transdisciplinary cooperation. These are not my cups of tea.

Regardless of the degree of theoretical embeddedness, the validity of arbitrary decisions classifying a given phenomenon, object or process as a social pathology (or its manifestation, genre, subgenre etc.) remains on the agenda. As aptly noted by (Laitinen and Särkelä 2019, p. 83), “[…] something is a social pathology if it is somehow ‘social,' and wrong.” This is, to put it ironically, a rather special marriage of two analytical categories: pathology and recognition of some assemblages in terms of social wrongs/evils. It goes beyond frames moral ougths-to-act and (*ad nauseam*) presented connections with politics and policies: “[…] there are *social* wrongs or evils of social practices, institutions, structures and processes in addition to narrowly moral and political one” (Laitinen and Särkelä, 2019, p. 84). In a word, broadly (or rather “stretchably”) understood social practices constitute the separation of spheres/domains of social pathologies (social evils or wrongs). They are at the same time an immanent component of the Manichean vision of the world, in which evil is, so to speak, functionally necessary, and once distinguished, it is subject to “naturalization” and reification. Of course, it is important who simply has stronger arguments and demonstrates greater proficiency in the art of fighting for preferences and their recognition as legitimized, but the question about the structural similarity of the definitions of social pathology constructed by indicating them remains unanswered. And finally, it may be worth tracing the processes of constituting social pathology on various “levels” of their “visualization”: starting from the ills that directly affect the wellbeing of individuals, groups, institutions, ending to—to use Zurn's (2011), p. 345) term—second-order disorders, i.e., conceptualizations related to the “content” of these ills. The second-order disorders block critical discernment of the situation, which, when subjected to analytical identification, appear as “[...] ideological recognition, maldistribution, invisibilization, rationality distortions, reification and institutionalized self-realization.”

The specification of second-order disorders is a sensible analytical procedure, because separating “reality” from “reflection” allows for the identification with full clarity of the processes that preserve, legitimize, and sometimes intensify, so to speak, the original (primary) social evils or social wrongs. I put these words in quotation marks not because I am suggesting any their distinct meanings, but rather because any reflection is, after all, part of that social reality. Without a doubt, if stronger actors/agents define situations as pathological, they are pathological in their consequences (especially for weaker or subordinated participants of social life), which, however, does not abolish the question of the “common conceptual structure.” More precisely: ignoring the attempt to answer this question increases the conceptual chaos and makes it difficult to theoretically organize the domain marked with the terms “social pathology” or “social wrong/evil.” Sure, one could argue that social reality simply is, but even if one accepts that it is *per se* amorphous, its ordering, usually or inevitably ideologically entangled, is a procedure that appeals to the intellect and the theoretical explanatory power of ideal-type constructions, to use Weber's [(1913) 2012] term. And even if one were to think consistently in terms of impression management, one needs to explain why some of the “pathology-like” images of social reality are more convincing. Sure, it can be assumed that “[...] the concept of pathology [...] adds a distinct layer of social wrongs or evils to the picture” (Laitinen and Särkelä, 2019, p. 87), but this is not the kind of theoretical one-sidedness that Weber had in mind, because it does not specify what a specific social wrong or evil. Or—to put it à* la* Goffman [(1974) 1986]—it does not indicate the frame to which these or other layerings refer.

Returning to Kavolis's idea, it is about phenomena that are destructive to the self and others, their social and cultural conditionalities. One can (and even should) argue about their ontological status, i.e., the level of their “anchoring,” whether they are first-, second- or third-orders, wondering whether their interpretation is characterized by organistic inspirations or borrowings, or perhaps they should be described as destructive in their consequences deviations from the norm. However, if such reflection is to “lead forward” in any way, and not constitute fodder for jugglers in the domain of language games, its preliminary function is to determine what actually paralyzes, limits, oppresses, harms actors, constituting an obstacle in realizing the opportunities or possibilities available in situations of action. The domain of social situations seems, I believe, to be the adequate platform or location for analysis. Its adoption makes possible to trace what and in what circumstances can have a destructive effect, whether individual varieties or “reservoirs” of pathology, in what constellations and with what destructive power they actualize their destructive potentials. Moreover, actors in situations of action, to refer to the findings of rational choice theorists, do not have to be understood as subjects striving to maximize profits and minimize costs, but rather strive to achieve states that are satisfactory to them. The social situation is a prism in which various influences, external and internal, present on this “platform” are refracted as more or less irresistible “offers” or “proposals” for shaping relations with the environment, also in the sense of interpretation and the possibility of activating the dispositions and predispositions of acting subjects. In this sense, any “offer” that can be subsumed under the banner of “social pathology” or “social evil/wrong,” is already prepared or predefined. For example, it can be presented as a single destruction related to alcoholism or economic exploitation, or as “drunkenness of the nation” or “unrighteous profit.” Sometimes it is perceived as a series of destructive “costs” for the self and others, when it is captured in categories of codependence, a completely irrational subordination of one's life to the actual (or rather reflected, in Cooley's understanding) needs of the alcoholic, as Beattie (2011) persuasively writes. Finally, it is conceived as a conglomeration of misfortunes, evil and destruction on almost all levels of social reality, to recall the impact of the idea implemented by some prohibitionists.

The main difficulty, I believe, is not that the phenomenon of social pathology is approached organistically, medically, normatively, in terms of social/public policies, critically or in any other way, because each of these approaches is a more or less successful attempt to analytically “pin down” the sphere marked as social evil or social wrong. Each of these “analytical spotlights” illuminates or generates not only partial views of this phenomenon (which is trivial in its obviousness), but also (and above all) offers alternative and non-trivial theoretical scenarios of its approach, emphasizing selected aspects of it. However, I am far from postulating some synthetic view in the capacity of the analyst, who, located in some theoretical (and ideologically sterile) panopticon, applies instruments from different theoretical toolkits. I am not interested in triangulating at the same time points of view and data in the name of constructing a substantive theory of social pathology. Syncretism is a somewhat pejorative term for this type of practice, and not because syncretism or eclecticism usually act repulsively on those interested in constructing theories, but because such a research practice would deepen the chaos, blurring differences in the name of unfettered anything goes. At the same time, as one might expect, it would give rise to endless disputes as to what, in what order, separately or in combination (and with what) should be applied in acquiring and systematizing knowledge about the phenomenon of social pathology. These debates concern also how to alleviate, mitigate, “cure” or combat social pathology, and prevent the emergence of its new forms that are the result or side effect of dealing with the previous ones. And finally, it would inhibit the sociological imagination that Mills (1959) appealed for when he searched for real connections between personal troubles and social issues.

### Critical theory and social pathology

A fundamental turn in thinking about social pathologies, social evils and social wrong is taking place, in my opinion, within critical theory, mainly through a somewhat restrictive but decisive move toward the analysis of pathologies of recognition. Honneth [(2000) 2007, (2007) 2009, 2010, 2014], refers to, *inter alia*, to the idea of social philosophy in the sense of Jean-Jacques Rousseau, understood as a diagnosis of social wrongs. He maintains that without the concept of normality it is impossible to judge the occurrence of social pathology. He emphasizes the importance of ethical criteria related to the idea of human self-realization, beyond the fringes of liberal interpretation focused on justice (also legitimacy). Moreover, he breaks with a somewhat static understanding of social pathology in terms of a fragmentary diagnosis in favor of its understanding as a dynamic, developmental and relatively autonomous process that rather resembles a progressive infection. The discussion around Honneth's proposal (including, among others, the positions of Hegel, Habermas, Adorno, but also Dewey, Fromm, Canguilhem) leads to, so to speak, expanding and rescaling the map of social pathology (even in terms of deviations from normalcy or normativity). It also provides a relatively clear distinction between first- and second-orders of disorders and the presentation of connections with social wrongs and social evils. This means repeating the question about what degenerates social life and to what extent it generates individual and collective distractions in the area of wellbeing [see especially Canivez, 2011; Celikates, (2009) 2018; Freyenhagen, 2015, 2018, 2019; Harris, 2019, 2021, 2022; Haslanger, 2020; Hirvonen, 2015, 2018, 2021; Laitinen and Särkelä, 2018, 2019, 2020; Neuhouser, 2023; Särkelä and Laitinen, 2019; Schaub, 2015; Smith, 2017; Thompson, 2019, 2021; Zurn, 2011]. If there is a kind of naturalism, it concerns the possibility of actualizing the potential of growth/development and stagnation (Särkelä and Laitinen, 2019; Särkelä, 2017), which characterizes particular social arrangements. If we are to approach it in this way, it is necessary to look decisively at sociocultural reality in terms of the structured process and ways of organizing experience proper to its actors/agents. The question is: whether and to what extent, within the “field of influence” of these or other assemblages, actors/agents are able to exploit the chances or opportunities available within social situations and (to restore the narrative à* la* Kavolis) are able to restrain or mitigate destruction in relation to self and others? It is also important whether and to what extent, in this constant movement from situation to situation, they retain a sense of agency. And it is not only about recognizing some behaviors in a situation as symptoms of embodied (although sometimes latent or hidden) evil or pathology, but also about seeing them in a broader plan, when the social becomes an inseparable component of a life-process.

Remaining within the framework of critical theory, it would be difficult to identify a “leading” or “dominant” interpretation of the phenomenon of social pathology. In his immanent critique of Honneth's findings, Freyenhagen (2015) meticulously demonstrates subsequent attempts to categorize (or operationalize) the term “social pathology.” The findings contained in “Pathologies of the Social” [Honneth, (2000) 2007] can be considered an outline of a scientific research program, exposing the basic areas of analysis, problems, or puzzles that must be addressed. These include, above all: the connections between social pathology and the concept of normality, ordering social pathology by ethical criteria, the developmental dynamics of social pathology and its own logic, and the anthropological interpretation of social pathology “as a deviation from an ideal” (Freyenhagen, 2015, p. 132–133). These are, in essence, “vectors” directing subsequent analyses and modifications of the theoretical approach to the phenomenon of social pathology. Addressing these analyses in terms of critical theory, according to (Freyenhagen 2015, p. 134–136), involves identifying three distinctive unit-ideas: (1) the connection of social pathology and normality “to rationality and its historical unfolding,” (2) “the process of deformation of rationality,” and (3) the emancipatory interest in overcoming it. Both the basic “vectors” of analysis and the way they are embedded within critical theory stimulate a revision of primary and secondary “operationalizations.” For example, the concept of self-realization, originally conceptualized as “the ideal of human self-realization” in connection with “ethical criteria,” is referred to the normative reconstruction of types of organized self-realization. This entails—as (Freyenhagen 2015, p. 151) notes—a conceptualization “in terms of detriments to individual wellbeing that is socially caused,” as well as a normative reconstruction of not only “social institutions involved in the reproduction of the existing social world, but also the countercurrents to it.” More broadly, reading Honneth's works and their critique, to refer also to other statements by (Freyenhagen 2018, 2019), encourages the creation of a kind of interdisciplinary analytical grid, divided into four analytically distinct tasks: “(1) symptomology, (2) diagnosis, (3) etiology, and (4) prognosis and therapy” (Freyenhagen, 2018, p. 16). Such a division of labor structuralizes the discussion around social pathology and, to a certain extent, forces the presentation of its connections with social ontology and the way it can be conceptualized in terms of “evidence, validation, and defense” (Freyenhagen, 2018, p. 19). The lack of a “final” interpretation is understandable, as it proves that we are dealing with a genuine theoretical puzzle.

The medical, organistic, evolutionary, or normative connotations associated with the concept of “social pathology” can be understood as aspects or layers of a broader life process. This means an explicit shift to the position of “the thick sense of social pathology.” On the one hand, it may tend to present *ad libitum* the presuppositional entanglements of particular approaches or theoretical positions. On the other (which is crucial), it abolishes or neutralizes the power of narrowing (“thin”) views, and in particular means breaking away from the organicist understanding of the society and the static approach to the individual–society relationship (Hirvonen, 2018, p. 10; Laitinen and Särkelä, 2019, p. 92 et seq.). Of course, this is not some kind of “liberation” from the sphere of limitations, constraints and influences located in the ontologically heterogeneous environment of action, which, in any case, could be described, following Fine (1984), as obdurate reality. Actors/agents are part of a larger functional whole, and its “dysfunctions” become significant for them (also in the sense of Luhmann's reduction of complexity) when they lead to stagnation or degeneration of their life processes. And it is not only a question of whether these “disruptions” are acute only at one moment or another, but also of whether they are relatively durable, transituational, and combined with relatively well-crystallized (social) habits [Camic, 1986; Caruana and Testa, 2021; Crossley, 2001; Dewey, (1922) 1930; Graybiel, 2008; Rayo and Becker, 2007], lifestyles (Chaney, 1996; Sobel, 2002), figurations [Elias, (1939) 2000], practices (Turner, 1994; Schatzki, 1996; Schatzki et al., 2001], or forms of life [Simmel, [(1904) 1997]; Jaeggi, (2014) 2018]. This “combination” means that the actor/agent operates in a predefined environment, subjected to the influences of the “field,” “socialized” in the sense of cognitive-emotional readiness to take on (or not) challenges in a situation of action, in a word, “profiled” in one way or another. And it depends on him/her, or more precisely: the relationship he/she develops with the environment, whether he/she will break through the “wall” of obdurate reality, e.g., by opposing secondary deviance on the part of significant others and the institutions of the justice system, or will he/she rather accept labeling, with all the staffage of transituational consequences, in terms, for example, of a sexist, a criminal, a marginalized person, inherited helplessness, a welfare queen, etc.

### Rahel Jaeggi's forms of life theory

Jaeggi [(2014) 2018, p. 41 ff.; see also Allen and Mendieta, 2018] focuses her analysis on those “ensembles of practices and beliefs” that turn out to be relatively durable and self-sufficient, and at the same time have a correspondingly strong or deep significance in shaping human life. From my perspective, it is crucial to analyze the circumstances in which these “ensembles” cease to function and prove “ineffective” in the face of disorders. This is not just about difficulties or troubles adapting to the environment, but also about disturbances in the sphere of interpretation.

The “ensembles of practice and beliefs” are transituational (even objective), transcending the sphere of the immediate, sporadic, or superficial, and are not isolated practices but function as “interconnected and interrelated practices.” Referring to Simmel's [(1904) 1997] analysis of fashion; Jaeggi [(2014) 2018, p. 50] states that forms of life are at the same time particular, i.e., tailored to a certain segment of social life, and that everyone in the situation of a certain actor/agent, or everyone in the position occupied by that acting entity, “should act in such and such a way.” Following Alfred Schutz (Schutz and Luckmann, 1973), we can say that we are dealing with the interchangeability of perspectives (including systems) of relevances, and—following Goffman (1983)—we can capture the functioning of forms of life in terms of structured interaction order(s). Such an association seems all the more justified because Jaeggi [(2014) 2018, p. 50–51] maintains that forms of life are in fact “nexuses of practices, orientations, and orders of social behavior,” both normatively marked and related to what is collective or supra-individual, components of “a normative social order with a claim to validity.” In a word, they are “instruments of adaptation to reality, are individual through the reality or matter they address,” constituting an inseparable component of problem-solving within a structured life-process. Such an approach, it should be added, enables not only addressing forms of life in terms of rightness, correctness or appropriateness, but also requires tracing the relationships between the whole and the parts (e.g., the relationship of the nuclear family with modernity—a broader form of life), as well as the relationships between the substantive and the contingent (e.g., lifestyles). It is not about recording simple causalities, but meticulous analysis of “overlaps and relations of influence, connections, associations, and relationships” [Jaeggi, (2014) 2018, p. 53]. And if tracking pluralization and transformation of forms of life is conceived as relevant, it is necessary ask about “different kinds of possible diversity” among them. It deals with the nature and scope of these transformations: from minor “discolorations” of meaning, through functional differences, to the constitution of alternative or competing functional and interpretative contexts in a pluralistic society.

Recognizing the modularity of forms of life inevitably leads to the question of what might disrupt their functionality and normative anchoring. At the heart of the controversy over these contexts of action is a disruption of what Jaeggi [(2014) 2018, p. 69–72] calls the relations of fit, whether in the sense of appropriateness “to the interpretive framework,” or “to the purposes of a nexus of practices.” Moreover, forms of life often “present a moment of inertia,” slowing down, mitigating or resisting human actions, constituting a pre-reflective component of the actors' experience. This is a kind of situational rudiment, often materialized and embodied (thing-like) in practices, institutionally codified, transformed into habits and practical routines, skills and competencies—various know-hows “sedimented” as implicit or explicit components tacit (implicit) knowledge [Jaeggi, (2014) 2018, p. 73–78]. The self-evident nature of forms of life, if only because of the dynamics of the situation, both underlies every interaction (in terms of taken-for-grantedness) and can itself be undermined when something does not function *comme il faut*. Questioning a specific habit or routine may not only lead to its “visibility” and reformulation in terms of reactivation or upgrade, but also sometimes force a “switch” to a different functional and interpretative context. An experienced police officer, who is identifying a suspicious group of people, is particularly careful, somehow upgrading previous habits and routines related to the role of a police officer. However, if he applies them to family members, neighbors or colleagues, then restoring self-evidence [or background expectancies, as Garfinkel (1967) would say] requires a “switch” to another, decidedly “non-uniform” functional-interpretive bundle.

Forms of life are open to change in both the could-be and should-be dimensions. They are not for someone or something, they are not good or wrong in themselves, they simply exist and have effects. This is the context of their application within the framework of problem- solving processes, in situations of action, decides which of the available “solutions” turn out to be appropriate or inappropriate [Jaeggi, (2014) 2018, p. 134–135]. In other words, forms of life, as “at once given and made” functional-interpretive sets, operate at the level of practical action as problem-solving strategies. The fundamental question concerns the circumstances in which certain forms of life cease to be “livable,” become “uninhabitable,” leading to a crisis in solving-problems process (and unmet needs). Their deficiency can be understood as a “functional” (pre-normative and interpretation-free) inability to solve a problem defined by a certain form of life or it can mean an “ethical-normative” inability to satisfy normative claims [Jaeggi, (2014) 2018, p. 153–154]. And since every solution to a problem has its “effects,” they inevitably also—as components of subsequent problem-solvings—require “appropriate” solutions. Consequently, if we do not reduce struggling with problems to a simple recipe of an one-shot solution, but to grasp them in terms of a broader context, the transituational continuity of experiences, it means that they become—as a result of successive layers constructed in situations of action—higher-order problems [Jaeggi, (2014) 2018, p. 163–164]. This “complexity” may mean a kind of “complication” based on an external cause (or simply contingency), which results in a shortage or lack of access to a certain resource (whether it is, for example, the lack of rainfall in an agrarian form of life, or a sinologist's poor knowledge of Chinese). In this sense, the potential crisis does not concern a particular form of life, it is a problem for it, as Jaeggi [(2014) 2018, p. 165] puts it. Each of these two exemplary situations, however, may become a problem posed with it, when the particular cause or concretization of contingency “encounter *internal shortcoming* of the constitutive practices and institutions of the form of life itself” [Jaeggi, (2014) 2018, p. 165]. For example, when no effort is made to collect water or when a sinologist makes no effort to improve his/her knowledge of Chinese and his/her knowledge of Chinese civilization is limited to ordering dishes in a Chinese restaurant. With yet another type the situation is when the source of crisis (or problematic) is immanent, that is when “the problems arise already out of the […] contradictions and the immanent fields of conflict implicit in the structure of the practices that constitute the form of life” [Jaeggi, (2014) 2018, p. 167]. In a word, what is posed with means going beyond strictly empirical references to the world. It should be understood in terms of conceptual (second-order) problems, which are based on deficiencies or shortcomings related to a specific form of life, the interpretation of practices and events within it, as well as in relation to other (alternative or substitute) forms of life [Jaeggi, (2014) 2018, p. 169–170].

The possibility and ability to shape forms of life, related to the possibility of reflection or insight in terms of emancipation in the sphere of practice, constitute the possibility of critiquing individual forms of life and assessing their rationality. Forms of life guide collective self-determinations and must be “corroborated” in subsequent (historically and structurally changing) situations. They have to connect what is found or determined with what is a historically developing horizon of expectations. Each of these situational versions directs a rational coping with problems in the life-process, taking into account ethical and functional norms proper to each of them. This is a process of learning *sui generis*, perfection in the art of experimentation in the name of finding functionally and ethically satisfactory solutions. This or that form of life turns out to be adequate or good insofar as it works as a strategy of action, or inadequate or wrong when appears to be a “collective practical deficit.” What is more, we are dealing with an irreducible pluralism of life forms, i.e., a multitude of situationally available solutions. Some of them turn out to be wrong or regressive “by their inability to deal with problems and crises” when individual problem constellations cannot be “deciphered,” perceived and placed in the spectrum of the rational learning process [Jaeggi, (2014) 2018, p. 315–318]. It is also, as Jaeggi [((2014) 2018), p. 319] states, “a pluralism of debate over the correct solution to the problem of successful form of life.”

## Final remarks

The positions of Kavolis and Jaeggi, seemingly distant from each other and rooted in decidedly different theoretical traditions, show the advantages of the heuristic that juxtaposes forms of life (or “related” moral systems/cultures) with the ways of actualizing the self-realization potential *resp*. limiting phenomena that are destructive to the self and others. Such an approach goes beyond the limitations associated with this or that social and political philosophy, especially liberal philosophy. It allows for recognizing phenomena outside the sphere of injustice, identifying previously poorly recognized or unrecognized maladies (e.g., threats of the Internet or global warming), setting the analytical crosshairs on phenomena that limit the potential for human flourishing (Neuhouser, 2023, p. 10 ff.; see also Harris, 2019, p. 47–49) or weakening “a presupposition for a good life among us” [Honneth, (2000) 2007].

I consider the idea of “asocial sociality,” identified by Laitinen et al. (2015), p. 8), to be both bridging and bonding in relation to the three leading narratives in my article. The meaning of this term is determined by the presuppositions of “an inbuild need for recognition” and universal “human tendencies toward the denial or rejection of recognition.” Recognition, articulated in terms of needs, demands and struggles, is a constitutive component of human existence. In this sense, the idea of “recognition,” or rather “intersubjective recognition,” is referred to “distinctively human psychological and social structures.” It also determines whether and in what extent they can “function well,” contributing to “the wellbeing and freedom of individuals, and for the moral or ethical quality of their relationships, characters, motives and actions” (Laitinen et al., 2015, p. 7). Such a clarification, from my point of view, has several important consequences. First, it “neutralizes” the somewhat optimistic tone of Axel Honneth's analyses, the focus on positive conceptualizations of structured recognition processes. To put it somewhat old-fashionedly, the consequences of intersubjective recognition can be (eu)functional, dysfunctional or non- functional. Secondly, this “functionality” is related to the moral “saturation” and ethical embeddedness of human actions, both in relation to the norms of “conventional” society and to particular normative standards in social situations. Thirdly, it draws attention to those components of power/authority that prove to be not only situationally, but also transituationally decisive in shaping the definition of a situation. It concerns both the vertical dimension of recognition, i.e., “between persons and norms or institutions,” as well as the horizontal dimension, i.e., “between persons” (Ikäheimo, 2015, p. 27–28). Fourthly, consistent “clarification” of this recognition-dependence requires analysis “[…] in terms of specific kinds of *psychological vulnerabilities and costs*” incurred by individuals aspiring to this or that form of recognition (Laitinen et al., 2015, p. 8). In other words, there is a connection between mutual recognition and individuals' motivation to recognize others. Just as there is a connection between mutual misrecognition and the rejection of recognizing others.

The analysis of recognition and pathologies of recognition become “thick” or transdisciplinary, transcending the framework of critical philosophy and critical social theory. It also transcends medical, biological, and psychiatric connotations, while simultaneously “bracketing” the organismic idea of human society. In my opinion, this does not necessarily have to lead to a complete abandonment of those concepts of social pathology that are founded on the idea of a social organism or the identification of “the shared conceptual structure of the pathologies without stressing the medical, biological or organismic aspect of the concept” (Laitinen et al., 2015, p. 9). These generalized discourses (organismic and critical) can be re-addressed “in terms of the reproductive disturbances in the process of social life” (Laitinen et al., 2015, p. 13). The characterization of these “disturbances” allows for a certain consolidation of the previous findings in terms of relatively permanent disorders that degenerate or reduce the potential of individuals and collectivities in terms of both welfare and wellbeing.

At the “origin” of any form of social pathology lies social wrong (social evil) or, more broadly, misdevelopment in the domain of the life-process. (Laitinen 2015, p. 46), bridging these “negatives” and social pathologies, distinguishes four basic aspects of orders of these phenomena. The first aspect concerns a certain failure embedded in social reality. The second deals with “distortions” affecting the experience and understanding of the “content” of social reality. The third aspect refers to second-order disorders as defined by Zurn (2011), i.e., distortions in the reflexive understanding of the components of the first two aspects. The fourth aspect deals with “blockades in the aspects of the social world which make it less receptive to reflectively formed critical insights” (Laitinen, 2015, p. 46). This typology proves all the more useful as Laitinen identifies three “three loci of disconnects” between the components of the four distinguished aspects. The first locus corresponds roughly to either Durkheimian anomie or refer to deficits in suitable socialization, making it difficult to act within the framework “the operative social reality” or sometimes exclude it (Laitinen, 2015, p. 48). The second locus refers to the systematic blocking of critical reflection of social actors/agents. As (Laitinen 2015, p. 50) argues, the disconnection between “[…] first-order contents and second reflection are a *necessary* aspect of social pathologies, but not the *sole aspect*.” This “not the sole” signifies a more “inclusive” (compared to Zurn's findings) view of social pathology. These are, of course, phenomena/events that testify to oppression and its “replica” in the form of common-sense (first-order) beliefs shared by victims. These are cases where the mechanism of “brute power/violence” operates in a way despite these or other beliefs related to a specific form of oppression. These are also cases in which first-order beliefs are perceived “as natural, unchangeable, are to be taken for granted, rather than created by social mechanisms.” And finally, certain social mechanisms can generate “problematic first-order beliefs,” blocking reflection at the second-order level (Laitinen, 2015, p. 49–50). The third locus can be found “between critical second-order reflections and the social world to be reshaped” (Laitinen, 2015, p. 46). The category of third-order disorders allows to indicate yet other “forces” disturbing the “effective social criticism,” preventively blocking of someone's motivations and capacity for action. This applies to quite a large group of phenomena: from self-censorship, through labeling, to disregarding someone's claims, considering them naive. And it is not just about discouraging signals of disapproval emitted by co-participants in the interaction, but also institutionalized practices/mechanisms of “pacifying in advance” and “pushing” outside the realm of legitimate claims.

In Laitinen's (2015) model, social pathology is mapped, and the basic components of this mapping are four aspects of “failures” and three loci of “disconnectedness.” In my opinion, this approach brings us closer to defining the “structural affinity” of social pathologies. Equally important is the establishment of new analytical trajectories, identifying key domains of disorders on which the researcher should focus. Social pathology is conceived as a complex or multilayered phenomenon. The inherent “interpenetrative” dynamics of “elements” located at different levels of disorder constitutes a genuine mystery box. It raises the question of the circumstances in which particular misdevelopments or failures become socially pathological and persist, transituationally “preserving” the collective and individual asocial socialities.

This way of thinking I also find in the “genealogically” different concepts of Kavolis and Jaeggi. The question of the nature of asocial sociability constitutes the fundamental heuristic of analysis in terms of non-contingent and transituational disturbances of the life-process, reducing or inhibiting the potential of individuals and collectivities. The sources/causes lie in the structured processes of organizing the experience of social actors. Paraphrasing Kavolis, potential pathogenicity (like normality) can be actualized when the acting subject is unable to face the demands of the situation. More precisely, this “inability” is not an interactional incident, a mishap, or bad luck, but stems from the inadequacy of ways of adaptation and generates “costs” that increase destructiveness “toward self and others.” In Jaeggi's concept, this adaptive “inability” is defined in terms of the weakening/inability of problem-solving. In both interpretations, it signifies, in its consequences, a relatively persistent tendency to minimize human potential. This is a kind of shifting the existential switch to the sidetrack of participation in social life. A return from this track to the mainstream “operative social reality” turns out to be structurally difficult and does not bring satisfactory results. To express this in terms of Randall Collins' ritual chains theory (Collins, 2004), it is the kind of participation in social life that pushes actors into the ruts of interaction rituals that lead to a transituational replication of losses in the “currency” of cultural capital and emotional energy. The characterization of social pathology is primarily an indication of the mechanisms behind “social processes of increasing deterioration” (Freyenhagen, 2018, p. 16). Although in this spectacle of pathogenesis the attention is drawn to episodes of pathological behavior, the term “deterioration” is the key to explaining them.

Characteristics of the processes of increasing deterioration usually includes, of course, the indication of symptoms of particular maladies. However, at the same time it should be oriented toward determining the causes/conditions of the process leading from particular “misdevelopments” to relatively permanent disturbances of the life-process. Kavolis emphasizes the connections between pathological behaviors and social and societal organization. It is not only a matter of determining whether any deviation contains specific symptoms “indicative of pathology,” but also of identifying what these symptoms might mean within specific sphere-of-life cultures. As components of these cultures, they define the dynamics of interaction, revealed as “moralities of the moment,” “moods,” and “energies.” They contain both indications for specific action and predefine its functionality (or dysfunctionality). However, an indispensable element of this functionality is reference to, as Kavolis puts it, moral cultures that demarcate right from wrong. And this moral anchoring of human activity, characterizing human actions in terms of responsibility and limitations, finds its ethical “alter” in the postulate of avoiding/limiting destruction toward self and others. The practical implementation of this postulate occurs within a specific moral culture which defines areas of evil, their etiology, and the possibilities of “struggles” for good. By analyzing forms of life, Jaeggi shows both the interactional dimension of could-be, related to the functionality, in a given situation, of the actions of social actors, and the dimension of should-be, i.e., the normative reference to the social order. These functional-interpretive sets are instruments for strategizing actions. They are adequate, fair, right, and good when they contribute to the achievement of individual aims and/or institutional claims. They become dysfunctional, wrong, and bad when they lead to a crisis in problem-solving and generate collective practical deficits. And if one is to look for an ethical justification, it is the postulate of limiting/changing such forms of life that do not prove to be strategies for effective and normative action in the problem-solving process.

Both in the concept of Kavolis and Jaeggi's theory, as well as in the specification of critical theory formulated by Laitinen, social pathology is not “assigned” to any of the levels of social reality. Malfunctioning within specific “segments” of social reality is a relational assemblage, a structured process incorporating components from various levels of disorders. And perhaps this heterogeneity (and its dynamics) makes social pathology appear as a multifaceted phenomenon. Sticking to the term “social diseases,” emphasizing the polymorphic nature of social pathology, seems to be insufficient. Each of these “diseases,” although it may cause an epidemic in a specific segment of the life-process, also attacks the “immune system” of the life-process in other dimensions. And it becomes part of the story of asocial sociability.

The concept of social pathology, when directly applied to the three narratives guiding my argument: critical theory, Kavolis's position, and Jaeggi's theory, focuses analysis on the life-process. This does not imply some terminological unity or the blurring of differences, but rather a convergence of analyses aimed at answering the question of the possibility of a good life. What unites such disparate narratives is, so to speak, a kind of normative individualism. It entails tracing the relationship between the sociocultural ideal of a good society (and its “emanations” in the form of forms of life or spheres-of-life) and the lived experiences of individuals. This is an ontology of becoming, the organization of social life, and the possibility of self-realization. It asks why the harmonization of the components of the social universe generates relatively persistent areas of disorder, inhibiting or limiting the possibilities of human potential under specific conditions. Such a theoretical specification can be conceptualized as a dynamic system of equations with many unknowns. But at least some of them are identified, mapping out the interplay of cultural patterns, the emergence of norms, power relations, interests, and human agency. The interest in social pathologies, social evils, social wrongs, or misdevelopments is a renewed attempt to identify, to paraphrase (Martin 2003, p. 44), the conditions we have not desired in order to achieve what we understand and want. This attempt takes the form of an essentially Kantian question about the possibility of synchronous “tuning” of limiting or minimizing destructiveness and strengthening the ability of collective problem-solving. And although diagnosis is the basis of analysis aimed at characterizing functionality, achieving “what we understand and want” requires normative anchoring and ethical evaluation.

It means direct addressing of the phenomena of social pathology (also social evils and social wrongs) in terms of social dynamics, collective social practices and their normative and ethical foundations. Such a “thick” perspective, while maintaining own immanent critical blade, requires, “social theory, and the primary objects of such critique are not individual actions but social practices and institutions” (Neuhouser, 2023, p. 11). The multiplicity of forms and varieties of social pathology does not exclude a relatively uniform ontology of the social world, or rather social life. Analogies with the animal world are inevitable, if only because of considering the possibility of survival and development in terms of specialization and coordination of activity, including self-organization. However, the social life of human beings goes beyond the framework of biological survival, both in the sense of “purely” human modes of orientation in terms of meaning and self-conscious life, as well as striving to realize functions defined in the non-material, spiritual sphere (e.g., freedom and recognition). In other words: the reproduction of society with actors/agents requires regulation by norms, conventions, and rules. Their validity is not limited to compliance with custom or utility, but to the realization of someone's potential through actions that are subject to evaluation in terms of goodness, rightness and adequacy. And this is a manifestation of self-determination, freedom to, and not only from, the activity of will shaping its actions with self-conception taken into account, harnessed in the modes of practices of collective life. This subjective anchoring of human action has its normative component, it constitutes a condition for cooperation with others (and in society), because it refers to the normatively and ethically marked will, consciousness and activity of others. At the same time, however, it constitutes a source of irremovable tensions between what is individual and supra-individual. These tensions are a component of the dynamics of social life. Their mitigating is often a purely mechanical or “animalistic” reduction of tension (usually through the aggression of frustrated individuals). It is also (and perhaps theoretically above all) a problem, the solution of which is achieved with the participation of ethical components present in social practices and institutions of social life. Sometimes it is achieved through reflection on the reorganization of the life-process in terms of extending the domain of freedom to and recognition from others (Neuhouser, 2023, p. 346–350).

The non-contingency of the structured process of social pathology is a theoretical puzzle that encourages reflection on the ontology of the social world, its addressing in relational categories. It means perceiving the connections between the “diseases of the social organism” and the degeneration and stagnation of the life-process. The characterization of this life-process primarily concerns illuminating the ways in which the relations of actors/agents with their environment are shaped and defining the conditions that make it difficult or impossible for the interaction to proceed as smoothly as possible. Some of them constitute a relatively permanent limitation, becoming negatives. The question of why some of these negatives are “promoted” to the role of social pathologies requires further analysis. It needs placing them on the research agenda together with the manifestations of social wrong and social evil, in order to better outline the trajectories of stagnation and degeneration. Such an analysis can undoubtedly strengthen the critical edge of sociological/social theory, and the refined analytical categories will allow for referring them to different types of societies.
